# Meanings of abortion in context: accounts of abortion in the lives of women diagnosed with breast cancer

**DOI:** 10.1186/s12905-017-0383-1

**Published:** 2017-04-05

**Authors:** Maggie Kirkman, Carmel Apicella, Jillian Graham, Martha Hickey, John L. Hopper, Louise Keogh, Ingrid Winship, Jane Fisher

**Affiliations:** 1 Jean Hailes Research Unit, School of Public Health and Preventive Medicine, Monash University, 553 St Kilda Road, Melbourne, 3004 Australia; 2 Melbourne School of Population and Global Health, The University of Melbourne, Melbourne, Australia; 3 School of Medicine, The University of Melbourne, Melbourne, Australia

**Keywords:** Breast cancer, Abortion, In-depth interviews, Qualitative methods

## Abstract

**Background:**

A breast cancer diagnosis and an abortion can each be pivotal moments in a woman’s life. Research on abortion and breast cancer deals predominantly with women diagnosed during pregnancy who might be advised to have an abortion. The other—discredited but persistent—association is that abortions cause breast cancer. The aim here was to understand some of the ways in which women themselves might experience the convergence of abortion and breast cancer.

**Methods:**

Among 50 women recruited from the Australian Breast Cancer Family Study and interviewed in depth about what it meant to have a breast cancer diagnosis before the age of 41, five spontaneously told of having or contemplating an abortion. The transcripts of these five women were analysed to identify what abortion meant in the context of breast cancer, studying each woman’s account as an individual “case” and interpreting it within narrative theory.

**Results:**

It was evident that each woman understood abortion as playing a different role in her life. One reported an abortion that she did not link to her cancer, the second was relieved not to have to abort a mid-treatment pregnancy, the third represented abortion as saving her life by making her cancer identifiable, the fourth grieved an abortion that had enabled her to begin chemotherapy, and the fifth believed that her cancer was caused by an earlier abortion.

**Conclusions:**

The women’s accounts illustrate the different meanings of abortion in women’s lives, with concomitant need for diverse support, advice, and information.

## Background

Contemplating or having an abortion can be a pivotal moment in a woman’s life; it is a topic on which there have been decades of research [1, 2]. A diagnosis of breast cancer is an existential threat that can profoundly change how a woman feels about herself and the world; this, too, has been extensively investigated [3, 4]. How might the two converge? How might a woman experience this convergence? Before contributing to this discussion from an Australian perspective, we give a brief introduction to the evidence on abortion and on breast cancer as discrete topics and introduce the two chief ways in which they are brought together.

### Abortion

The global annual abortion rate has been estimated at about 29 abortions per 1000 women aged 15–44 years [5]. One in four Australian women will have an abortion during her lifetime [6]. Abortion is sought by women at all ages and stages of life, with diverse social and personal circumstances [7, 8]. The reasons women give for seeking or having an abortion are usually complex and contingent, taking into account their own needs, a sense of responsibility to existing children and the potential child, and the contribution of significant others, including the genetic father; often the decision is made because the woman is not ready to be a mother or because another birth would be detrimental to her existing children [2, 9, 10]. Women who have contemplated or undergone an abortion may construct it as a difficult but necessary solution to a problem, as did women interviewed in Australia [11]. Similarly, Swedish women considered abortion “a painful necessity” [7].

A woman can experience her abortion as a relief, a source of distress, a minor disruption, or a reason for guilt, separately, sequentially, or in combination [7, 12]. An analysis of counselling needs assessment forms completed by more than 5000 women in the United States of America (USA) found that most women expect to cope well after an abortion [13]. Many women experience only positive emotions such as relief in the year after their abortions [14]. A longitudinal study in the USA found that women’s emotional intensity decreased in the three years following abortion and that almost all women continued to think that the abortion was the right decision for them [15]. Some women who hold strong anti-abortion views nonetheless decide that their situation is exceptional, seeking an abortion while maintaining their opposition to abortion in general [16]. Holding anti-abortion views is possibly an element in post-abortion guilt and other negative emotions [16].

Women’s feelings about their abortions can be influenced by public objections to abortion, especially by protesters at clinics where abortions are performed [17], although these feelings may be short-lived [18]. National newspapers in Britain were found to contain adverse representations of abortion that rarely included the perspectives of women who sought abortions [19]. The researchers noted that it was rare for abortion to be framed as “a positive and legitimate choice” and concluded that the media contribute to the stigmatisation of abortion and the discrediting of women’s reproductive decisions [19]. Public attitudes to abortion are highly contextualised; approval or disapproval depends on what is known of each woman’s circumstances [20]. Discussing abortion detached from its context can, therefore, result in misleading conclusions about public opinion. It has been argued that the stigma attached to abortion arises from its perceived enabling of women’s transgression of three idealisations of womanhood: perpetual fecundity, the inevitability of motherhood, and instinctive nurturing [21].

Australia has nine sets of laws (Commonwealth, state, and territorial) concerned with abortion, varying from abortion provided as part of reproductive health care to abortion restricted to instances in which it can be established that continuing the pregnancy poses a serious risk to the physical or mental health of the woman [22]. In contrast to the USA, where attitudes to abortion influence how citizens vote, it appears that Australian voters do not display a voting pattern associated with opinions on abortion [23].

### Breast cancer

Excluding cancers of the skin, breast cancer is the most common cancer diagnosed in women in the United States [24], Australia [25], and the United Kingdom [26]. Breast cancer brings with it a threat of death; successful treatment does not eradicate fear of recurrence [27]. Treatment for breast cancer can include surgery such as mastectomy and removal of lymph nodes, radiation therapy, and chemotherapy. These treatments are often harrowing, with difficult side-effects including hair loss, nausea, fatigue, and pain. Many women are left with permanent scars and functional damage. Literature and autobiography convey the profound meaning of breast cancer in a woman’s life [28–30], and researchers continue to accumulate evidence of the psychosocial aspects of breast cancer, both “objectively” and from women’s perspectives [31–36].

When breast cancer is diagnosed in women of reproductive age, breast cancer, fertility, and reproductive health are inter-linked in complex ways that have immediate and long-term consequences [37, 38]. Caution has usually led clinicians to advise their young breast cancer patients to delay conception for at least two years after treatment [39]. However, a population-based study conducted in Australia found that women who conceived six months after the conclusion of treatment did not have reduced life expectancy in comparison with women who did not conceive or conceived later [40]. Breast cancer treatment can diminish fertility by inducing or hastening menopause [41], and the often-recommended five years of endocrine treatment usually results in ovarian ageing in addition to deferred reproduction [42]. Young women diagnosed with breast cancer want clinicians to cure them or at least extend their lives as long as possible, but they also want their doctors to communicate fertility options and avoid assumptions about each woman's fertility desires [43]. Young women diagnosed with breast cancer have had diverse reproductive experiences before diagnosis and vary in their plans and hopes for motherhood [43].

### Abortion and breast cancer

#### Breast cancer diagnosed in pregnancy

Research on abortion and breast cancer deals predominantly with women whose diagnosis is made during pregnancy and who might be advised to have an abortion before chemotherapy can begin. Drawing on population data, it was found that gestational breast cancer occurred in 6% of breast cancers diagnosed in women under 45 years of age who lived in Australia; two thirds of these were diagnosed postpartum [44]. Therefore, only 2% of women diagnosed under 45 were pregnant at diagnosis. The same population data revealed that women diagnosed during pregnancy were more likely than not to have an abortion [44].

A diagnosis of breast cancer during pregnancy presents problems to women and their clinicians. Women confront difficult decisions about the life of the fetus, their own life, and what it might mean for their families [45]. The diagnosis can cause severe emotional distress that can last for years, especially if, among other things, women are advised to have an abortion [46]. When 17 women in Australia were interviewed about their experiences of health care after being diagnosed with breast cancer during pregnancy, they reported particularly disliking the insistence by some clinicians that abortion is the only rational response to cancer [47]. Interviews with another 15 women in Australia revealed their priority as staying alive, especially for the sake of their existing children, but that they longed to bring their fetus to term [45]. This was echoed in women pregnant with their first child who yearned to have the baby but wanted to ensure they were alive to mother the child [45]. Among the 15 women interviewed, only one had an abortion, for which she expressed regret even years later [45]. Because a diagnosis of breast cancer during pregnancy presents the clinician with an ethical dilemma in reconciling the health of the mother and of the fetus [48], women may be given equivocal advice, thus making their decision even more difficult [45].

Guidance for decisions is not unequivocal, however. Recent evidence indicates that cancer treatment regimens do not necessarily harm the fetus and that abortion or early delivery may not be necessary [49]. The effects of cancer treatment (and thus the nature of treatment) depend on the stage of pregnancy, with greater risk to the fetus in the first trimester [50]. A retrospective analysis of biological features and treatment of 38 women consecutively diagnosed with breast cancer during pregnancy (*number [n]* = 21) or while lactating (*n* = 17) at one Italian clinic found that all 6 of the women diagnosed in the first trimester had an abortion, although an option for continuing the pregnancy had been discussed [51]. The remaining 15 pregnant women had conservative surgery during pregnancy and had no local recurrence within two years [51]. Five women received chemotherapy during the second and third trimesters with no complications for mother or baby [51].

#### Abortion as a cause of breast cancer

The second—discredited but persistent—association between breast cancer and abortion is that abortions cause breast cancer. The paper most commonly cited is Daling et al. [52], who conclude that their “data support the hypothesis that an induced abortion can adversely influence a woman’s subsequent risk of breast cancer”. (Their request for caution and more data is often overlooked.) The claim is based on retrospective data subject to recall bias; women with breast cancer are more likely than those not so diagnosed to recall and admit to having had an abortion as they seek an explanation for this existential challenge. Nevertheless, Googling “abortion causes breast cancer” yields many non-peer-reviewed papers and even more websites asserting this causal connection and using it to support arguments against abortion.

Claims that abortion causes breast cancer are not sustained by extensive analysis of large, pooled, international sets of prospective data, performed, for example, by the Collaborative Group on Hormonal Factors in Breast Cancer [53]. This group concluded that abortion, whether spontaneous or induced, is not associated with an increase in a woman’s risk of developing breast cancer [53]. Data from the California Teachers Study led to a similar conclusion [54], as did Australian results from the Melbourne Collaborative Study’s cohort of 24,000 women [55].

The effects of such misinformation about abortion is evident in some women’s beliefs. In one recent USA survey, 6% (4/67) of women were found to believe that abortion causes breast cancer [56]. When women in Australia were asked about what they thought had contributed to the development of their own breast cancer [57], or surveyed about their beliefs about breast cancer causes and risk factors [58], neither research instrument included “abortion” as an option.

### Aims

In this paper we report on an outcome of research conducted with women of reproductive age who had been diagnosed with breast cancer. During in-depth interviews focused on fertility and motherhood, a few women spontaneously raised the topic of abortion. The aim of the original project was to inform and enhance immediate and long-term supportive care for women diagnosed with breast cancer during their reproductive years (Kirkman M, Doran F, Graham G, Apicella C, Keogh L, Winship I, Hopper J, Stern C, Hickey M, Marigliani R et al.: Function, appearance, and womanliness in 886 women’s reflections on their bodies after treatment for breast cancer: A 887 qualitative investigation using a population-based sample, under review). Our aim in undertaking the analysis reported here was to contribute a contextualised and nuanced understanding of the diverse ramifications and meanings of abortion in women’s lives. Specifically, we sought to understand the meanings of abortion to women who had been diagnosed with breast cancer before the age of 41 years.

## Method

We recruited women from the Australian Breast Cancer Family Study [59]. The group we called the *Historical Cohort* comprised women diagnosed with breast cancer, in the State of Victoria, Australia, from 1994 to 1999, when they were aged 18–40, who had completed a 10-year follow-up questionnaire in 2009. Women in the *Contemporary Cohort* had been diagnosed with breast cancer in Victoria in 2009, when they, too, were aged 18–40 and had completed a baseline questionnaire in 2010–2011. From September 2011 to March 2012, staff of the Australian Breast Cancer Family Registry invited randomly-selected eligible women in the Historical Cohort to participate, having first ensured that there were no notes in their record that they did not want to be contacted or that it would be inappropriate to do so. Interested women returned permission forms which were forwarded to the researchers who sent information packages to the women, who could choose to make an appointment to be interviewed. One follow-up call was made to women who did not return permission slips. The process was repeated for the Contemporary Cohort, with invitations sent January-March 2012.

Fifty women volunteered and were interviewed after giving informed consent in writing or (for most telephone interviews) orally. Interviews (conducted by either the first or third authors) began by inviting women to talk about their experience of being diagnosed with breast cancer, and emphasised their fertility-related experiences, expectations, and reflections. Discussion was also directed towards their children or their childlessness, their partners, sexual relationships, concerns about health and recurrence, and advice women might offer to other young women being diagnosed with breast cancer and to their clinicians. The topic of abortion was not raised by the interviewer. Given the option to be interviewed in person or by telephone, most women chose the telephone. Interviews lasted a mean of 45 min, with a range of 21 to 82 min. All interviews were audio-recorded, with permission, and fully transcribed. Identifying details on the transcripts were changed or deleted; women were invited to choose their own pseudonym. We used interpretative phenomenological analysis [60], informed by narrative theory [61, 62], on our transcripts to gain insight into women’s experiences of the aftermath of breast cancer. Applying Bruner’s [61] *narrative mode of thought*, which represents the human need to find meaning and explanation in the vicissitudes of life, allowed us to take account of context and intention in women’s accounts.

### The present study

Women in the original research who spontaneously introduced the topic of abortion were included in this analysis. In Victoria, abortion is a woman’s choice up to 24 weeks’ gestation; after 24 weeks, two medical practitioners must agree that abortion is appropriate [22].

When a few women described their experiences of abortion and what it had meant to them, sometimes fairly briefly, we thought that this unsought outcome called, post hoc, for a case study approach. The case study method integrates well with narrative theory because both acknowledge context and meaning and both can give insight into reflection in individual lives [63–65]. We understand *case study* not in the medical sense, where a doctor transforms an illness narrative into a case for reporting an interesting symptom or disease course, but, in Radley and Chamberlain’s [66] words, as representing individual “discursive agents” who shed light on the ways in which meaning is influenced by and constructed within the personal context—in this research, of the experience of abortion. Radley and Chamberlain’s [66] discussion of the value of the case in health psychology is consistent with much of the writing on illness narratives [67, 68]; we take a case to be a more concise representation of the discursive agent, with less emphasis on plot and other characteristics of narrative analysis.

In our analysis, the women’s transcripts were read several times (by the first and last authors) to identify all that they said or implied about their experiences of abortion. Our endeavour was not to assess women’s psychological states but to identify what abortion meant to them, especially in relation to breast cancer. Having summarised each woman’s account, we discussed and reflected on them in the light of evidence in the research literature.

## Results

Five of the 50 women interviewed described their experiences with abortion: Gaelene, Fiona, Katrina, Karen, and Kristina. All but Karen, who came to the university to be interviewed in person, chose to be interviewed by telephone. Their ages at interview ranged from 40 to 52 years and at the time of abortion or potential abortion 31–40 years. Details are in Table 1. In contrast to the other four women, Kristina’s first language is not English.

**Table 1:** Women’s ages at interview, breast cancer diagnosis, and (potential) abortion

Pseudonym	Age at interview	Age diagnosed	Age at (potential) abortion	Historical or Contemporary cohort
Gaelene	41	27	34	H
Fiona	40	37	40	C
Katrina	52	35	34	H
Karen	46	32	32	H
Kristina	40	37	31	C

Gaelene, Katrina, Karen, and Kristina were recalling abortions that happened at least nine and up to 17 years before they were interviewed; Fiona was pregnant at interview and told of having to contemplate an abortion for that pregnancy. Each woman understood abortion or its prospect to have played a different role in her life. Gaelene and Fiona emphasised the reasons for undergoing or contemplating an abortion; Katrina, Karen, and Kristina (whose alliterative pseudonyms occurred by chance) reported meaningful connections between their abortions and breast cancer.

### Gaelene: abortion for ill-timed conception, unrelated to breast cancer

Gaelene has three children, two of whom were born before her diagnosis of breast cancer (at the age of 27) and one after treatment had ended. She separated from the father of her first two children not long after her diagnosis; he did not support her but she did not attribute the break-up to her cancer. The father of her third child left her in acrimonious circumstances during her pregnancy; legal consequences were continuing at the time of interview. Gaelene would have welcomed a daughter after three sons and even embarked on assisted reproduction at the end of her cancer treatment but, when she conceived spontaneously with a short-term partner, she did not feel that it was the right time to have another child and had an abortion: “I wasn’t with the person very long, and my youngest wasn’t very old. I think he might have been under two.” Gaelene did not dwell on her abortion experience, despite having come to believe (at the age of 41) that she will have no more children.

### Fiona: abortion considered because of conception during cancer treatment

Parents of two children, Fiona and her husband had “finished having our family” when she was diagnosed with breast cancer at 37. Three years later, when she was still taking tamoxifen as part of her cancer treatment and assumed that her menopausal symptoms precluded conception, Fiona was shocked to discover that she was 23 weeks pregnant. Knowing that “you’re not supposed to get pregnant on tamoxifen,” she was concerned that the drug might adversely affect the fetus and that the pregnancy might be detrimental to her health: “We Googled straight away, and when you go onto the internet, it’s all terrible. … Birth defects are common in all the tests that are done on animals.” Fiona and her husband considered abortion; he, in particular, “wasn’t comfortable with putting my life at risk. … I couldn’t speak that night. I think I was upset and shocked.” However, her oncologist was “quite reassuring”, as were her GP and obstetrician, that her health was not at risk and that the fetus gave no sign of abnormality. “If there was nothing wrong, we were very keen to go ahead, and we couldn’t believe it. We were in shock. … I kept thinking, ‘We’ve got too many children!’” Fiona was 28 weeks pregnant at interview and “excited” about having a third child. Nevertheless:

“I still say to Rob, ‘I hope everything is okay’. But the obstetrician keeps saying it’s all just a normal pregnancy now. Although I have to go and see a heart specialist on Friday. There was a few things that he wanted me to do after having chemo and radiotherapy to make sure I can go through labour and that my heart hasn’t been affected.”

### Katrina: abortion recast, after cancer diagnosis, as a life-saver

At the age of 34, Katrina had two children when she conceived again. “Issues going wrong” in her life led her to make the “difficult” decision to have an abortion. Within a year, Katrina was diagnosed with breast cancer. The abortion has come to play a beneficial role in Katrina’s breast cancer story because she “was told by the medical people that fortunately that did happen, that things were wrong and I did terminate, because that would have possibly made the cancer a whole lot worse”. Not only would the pregnancy have caused rapid development of the cancer, according to Katrina, but lactation would have masked the tumour, thus delaying diagnosis. Rather than feeling robbed of future children, Katrina concluded that, “I had my two healthy little girls. … I was lucky; I felt I was lucky”.

### Karen: abortion necessitated by breast cancer diagnosis as a continuing source of grief

Karen was in her first trimester with her second child when she felt a lump in her breast that was diagnosed as malignant. She was 32 years old. Karen wanted to delay chemotherapy until the fetus was viable, but was told that it would reduce her chance of survival. She and her husband decided that she would have an abortion, for the sake of her own health and to ensure that their 4-year-old son had a mother. Karen was “devastated”. She thinks her husband “saw the here and now: What I have now is a wife, and I have a son. To him, the baby wasn’t a baby until it was born”. In contrast, Karen “felt very strongly that, if I don’t protect that unborn child, then who is there to do that? … I felt very selfish choosing my own life. … I still have those emotions.” Because Karen “was so emotionally overwhelmed” at the time, she described repressing her feelings about her abortion until she had finished her cancer treatment, when she became able to consult a psychologist about the abortion, “to get a better perspective and, I suppose, to feel better about myself”. Karen eventually had two more children, saying of her time since diagnosis: “the loss of the child is definitely the greatest thing, and not having been able to fulfil that life is huge. So the need to have more children was definitely paramount”. The story of having an abortion to save her own life 14 years earlier dominated Karen’s account of breast cancer, and she wept while telling it.

### Kristina: abortion as a causal explanation of breast cancer

Kristina had two children who were in primary school when, aged 37, she was given a diagnosis of breast cancer. Kristina’s story was one of being misunderstood, of being poorly cared for and about, and of profound unhappiness with the ramifications of breast cancer and its treatment, including that she had gained 25 k. Her diagnosis was delayed when her concerns were dismissed; she had told doctors that that “Something might be wrong. But they all said it’s fine.” When she was finally diagnosed, Kristina had just finished a university degree. She reported saying to her doctor, “Well, this is my time to shine and go and find a job, and it’s just taken away from me”. Her expectations of support from her husband were low; she said that, after her diagnosis, “He was supportive. Yes, I can say that he didn’t leave me. Lots of men just disappear. So he was kind of supportive.” She felt she experienced “lack of continuous support” and insensitivity from her healthcare providers; for example, “the nurse sent me to the chemo ward straight away. It was like another slap; didn’t even ask me was I ready or not to see all of those bald people with fake, and, you know, pale faces.” At every turn, Kristina was “scared”, “terrified”, or “shocked”. She continued to consult different doctors after her diagnosis, saying, “I didn’t trust any more anybody”. When her psychological problems were recognised, she found the assistance she was offered to be unhelpful: “I have my psychologist, but you just keep telling the story. So, I don’t know, it’s not physical or direct help, practical help”.

Kristina appeared to be trying to find an explanation for her suffering and had concluded that breast cancer was caused by an abortion that preceded diagnosis by six years: “In my research I read that that could lead to breast cancer later in life, if you have a termination. … If I could go back, I would keep that child. God save me and forgive me.” Kristina had not been ready to have a third child, “Because I was in a different country with no [social or financial] support at all, and I’d thought that it’s not the right time.” “Immigration issues” had led to postnatal depression after the birth of her second child, and then she would have had three children under the age of four. Furthermore, her husband showed no interest in the prospect of another child, “and that’s another thing why I chose not to have it, because … that is also sad for the child, to have a father doesn’t really want him.” Moved by Kristina’s suffering, the interviewer offered to give Kristina evidence that there was no causal association between abortion and breast cancer, but Kristina declined, adamantly asserting the logic of the causal connection:

“Because I was already eight weeks pregnant, so the whole body started to change. … I was breastfeeding for quite a long time, so [my son] was just off from breastfeeding in November, and then I got pregnant the next June. … So if you get pregnant, you get kind of, you know, rounded, and it’s started to grow, and I think it’s something, because I had ductal carcinoma, so, you know, in the breast duct. … It might be something stayed there or didn’t clear up. … That’s my opinion.”

## Discussion

The accounts from these five women demonstrate the complexity of context and the variability in life circumstances that have been found to be crucial in understanding abortion [20]. They also reveal the different ways that abortion can be given meaning in women’s lives and how that meaning can change over time. Although each woman is describing her experience of abortion while telling her story of breast cancer, the women do not all fit neatly into the two previously reported links between breast cancer and abortion: diagnosis in pregnancy and abortion as a cause of breast cancer. Even in this small group of women we can identify diverse attitudes and meaning-making.

Despite this contribution to the literature, we acknowledge limitations. What we learnt about women’s experiences of abortion in the context of breast cancer was disclosed spontaneously; there would be great benefit in investigating this topic systematically. Furthermore, we were resourced to undertake research only with women able to communicate in English.

Abortion has been seen as a metaphorical “life saver” for women who feel that it is the wrong time to have a baby [11, 14, 69] but, as Katrina demonstrated, it can also be constructed as a literal means of saving a woman’s life. Katrina explained her early diagnosis and treatment for breast cancer as brought about by her decision to have an abortion rather than continue her pregnancy. According to Katrina, comments from her medical specialists helped her to understand her experience in this beneficial way; people do not construct life narratives in isolation but in interaction with significant others, influenced by the discursive environment [62, 70]. Despite two such challenging events—abortion and breast cancer—in the space of a year, it was still possible for Katrina to describe herself as “lucky”. In narrative terms, she had found a consoling plot [71, 72]: a way of interpreting the vicissitudes of life that gives them meaning and makes them bearable.

It is frequently found that women can make a decision to have an abortion without regret or distress [14]. Like Katrina, Gaelene had decided to abort, as women so often do [2, 10], because of other circumstances in her life that made it difficult to add a child to her family. Gaelene had sought help at a fertility clinic before conceiving spontaneously, which could invite the assumption that she would be distressed. However, she gave no indication of regretting her decision (consistent with the attitudes of women in other research [12, 14]) and it played no great role in her account. Memorable events, including abortion, can become the pivot of a life story (its whole meaning), the focus of a more specific narrative, or, as in Gaelene’s case, no more than a footnote to a narrative constructed around other events [73]. The full range can be identified in these five women.

When a woman is advised to have an abortion after a diagnosis of breast cancer in the first trimester of pregnancy, it can seem like a choice between her own life and the life of her baby [45]. As Karen revealed, saving oneself can be experienced as selfish, with guilt accompanying grief in her emotional response to the decision. Although Karen said that her husband wanted to save her life, she implied that he abetted her selfishness. Karen contrasted her own concern for the unborn baby with her assessment of her husband as caring only about the present, as being unable to see the fetus as a child. In the meaning Karen constructed from her circumstances, her husband’s care for her was inadequate compensation or justification for his lack of care for their child. Karen’s anguish is complicated by her recognition that her four-year-old son needed his mother; she did not imply that he deserved any blame for her quandary. Her continued distress is consistent with what has been found in other women advised to have an abortion after a diagnosis of breast cancer [46].

Even when women have decided that they want no more children, an unexpected pregnancy will not necessarily be rejected; the meaning of ‘unintended’ in relation to pregnancy is ambiguous at best [74–76]. Some women will welcome the pregnancy and be glad about the surprise (while simultaneously being discombobulated by the disruption to their plans). This was the case with Fiona, who had reason to fear that her continuing medication for breast cancer would be toxic to the fetus and that pregnancy would adversely affect her own health. She and her husband assumed that an abortion would be necessary; Fiona was told by her doctors that it would not. There is no indication in her account that she was under any pressure from her doctors to continue the pregnancy; the story she told was of clinicians who responded to her questions and concerns and reassured her. Fiona did not experience the distress of being advised to abort a wanted pregnancy (however unexpected), instead being assisted to manage it with care. Her throw-away remark about a need to keep watch on the health of her heart is an indication that her doctors were aware of potential problems and taking steps to avert or manage them.

The need to explain misfortune, to find a cause of calamity, is a powerful human drive and the foundation of many narrative plots [77, 78]. Not everyone, of course, asks “Why me?”, but those who do may find explanations in their own transgression, as did Kristina. There are powerful religious narratives of sin and punishment available to believers that, when combined with anti-abortion activism, provide a ready explanation for the chastisement of breast cancer. (This may lie behind the conclusions of retrospective studies of abortion and breast cancer, such as Daling et al. [52].) Kristina’s account of her breast cancer story presented her as a victim: of circumstances, of thoughtless doctors and nurses, of painful medical procedures, and of her husband and family. Breast cancer was the culminating punishment, and only for this was Kristina able to find a cause that she could understand. Clinging to that explanation appeared to give her the comfort and certainty lacking from the other indiscriminate cruelties of her world. Kristina’s story is a very fine example of the significance of personal meaning and the need to justify one’s actions or to demonstrate that unjustified actions have consequences [79].

## Conclusions

The accounts from these five women serve to illustrate the very different meanings of abortion in women’s lives, with concomitant needs for diverse support, advice, and information. Even so, the meanings they derive from their experiences and the way they construct their accounts are consistent with evidence of women’s diverse experiences of abortion in countries in which safe abortion is available. In choosing to identify our approach as drawing on the case study model appropriate to health psychology, we acknowledge that it is underpinned by an ethic of care [66] relevant to psychologists and medical practitioners. Care for women being treated for breast cancer is usually provided by multi-disciplinary teams that include surgeons, oncologists, breast care nurses, and allied health professionals. Not all members of such teams will feel equipped to inquire about sensitive aspects of women’s reproductive health. Our results suggest that being alert to the complex ramifications of pregnancy and abortion should be included in communication training, an endeavour recognised to be of increasing importance for those providing cancer care [80]. Perhaps the most salient lesson to be learnt from these women is that we cannot assume how a woman will feel about a pregnancy or an abortion, even in relation to such a significant event as a diagnosis of breast cancer, nor what decision she will make. Those who would like to know how women feel and what they think might well take up the challenge made by Harré and Secord [81] decades ago: “Why not ask them?”

## Abbreviations

N: Number
USA: United States of America
